# Impact of the War in Ukraine on the Ability of Children to Recognize Basic Emotions

**DOI:** 10.3389/ijph.2024.1607094

**Published:** 2024-05-13

**Authors:** Oleksandra Loshenko, Petr Palíšek, Ondřej Straka, Michal Jabůrek, Šárka Portešová, Anna Ševčíková

**Affiliations:** Faculty of Social Studies, Psychology Research Institute, Masaryk University, Brno, Czechia

**Keywords:** children, emotion recognition, emotion intensity, emotion recognition accuracy, war

## Abstract

**Objectives:**

This study assessed emotion recognition skills in school-age children in wartime conditions in Ukraine.

**Methods:**

An online survey based on the concept of basic emotions was administrated to a sample of 419 schoolchildren from Ukraine and a control group of 310 schoolchildren from the Czech Republic, aged 8 to 12.

**Results:**

There is no difference in judging the intensity of anger and fear by Ukrainian children, compared with the control group. There is no evidence that the emotions of anger, fear, and sadness were better recognized in the Ukrainian group. Children from Ukraine were better at recognizing positive emotions than Czech children.

**Conclusion:**

Increased risks of threats and wartime experience do not impair the accuracy of identification of emotions like fear or the assessment of intensity of basic emotions by children who experience war in Ukraine. Still, it is important to continue studying the long-term consequences of military conflicts in order to deepen the understanding of their impact on human mental functioning.

## Introduction

Being able to recognize emotions is an important characteristic of normal social functioning. In childhood, emotion recognition skills are one of the basic factors in forming proper social interaction, healthy communication in relationships, and subsequently healthy psychosocial development, increasing in researchers’ interest in this topic [1, 2]. However, most existing studies focus on the typical processes of children’s emotions recognition, while the social and environmental contexts are considered to a lesser extent. Hence, there is a significant literature gap regarding hostile environments in terms of their impact on a child’s ability to assess and identify different emotional states of other people. At the same time, the existing studies have not considered the context of war as one of the most traumatic environmental factors.

Nevertheless, several earlier studies show a relationship between the ability of younger individuals to recognize emotions and the level of threat and traumatic experiences in childhood [3–5]. In particular, according to Pollak et al., children who lived in a threatening and abusive environment recognized a lower percentage of emotions (59%) than well-off children (66%) [3]. That is, an increase in the danger of the environment might lead to deterioration in recognizing emotions.

Specifically, the War in Ukraine upended the lives of the children living in the region in a plethora of ways hardly imaginable to those living in peaceful conditions. Growing up in wartime entails disruptions to normal development in multiple areas [6]. Therefore, this study focuses on the impact of war-related trauma on the ability of Ukrainian school-aged children to recognize basic emotions.

### Ability to Recognize Emotions

Researchers attribute emotion recognition to a type of social cognition, namely, a person’s ability to perceive, process, and interpret social information [7]. Slušnienė notes that a person’s ability to capture, identify and analyze emotions is a sign of sustainable personality development [8]. Adolphs indicates that humans prefer using physiognomy in recognizing emotions, and early processing of faces is one of the functions of the cerebral cortex [9]. In particular, according to Guarnera et al., younger children use facial information to recognize emotions such as happiness, anger, sadness, surprise, or disgust [10].

The crucial role of emotional recognition in social interaction is integrated into Crick and Dodge’s social information processing model [11]. According to the model, social information processing begins with the situation encoding stage - before assessing the situation and taking the necessary actions, children find out what exactly happened; that is, they evaluate the context. Similarly, Tyng et al. identify the importance of recognizing emotions in their direct influence on the formation of adaptive behaviors through learning [12]. These findings are supported by Halberstadt et al., who argued that children’s ability to interpret emotions is highly significant for their healthy social and cognitive development [1].

### Effects of Threatening Environment on the Perceived Intensity of Emotions

A child can recognize basic emotions such as joy, anger, sadness, fear, surprise, and disgust [13]. Still, Garcia and Tully point out that the accuracy of emotion recognition increases with age, meaning that mild and moderate emotions are better recognized in later childhood [13]. In addition, the recognition of emotions of happiness and anger does not require their sufficient intensity, while the emotions of sadness are recognized by children better if these feelings are clearly expressed [13].

Earlier studies also report the universality of emotion recognition in childhood. For example, younger children are generally poorer at interpreting emotions than older children [14], which is confirmed by Gao and Maurer also show that emotional recognition performance improves with a child’s life experience [15]. Mancini et al. also examined changes in the ability of normally living children to recognize emotions from facial expressions as their age increased [16]. In addition, positive emotions are recognized more clearly by children of all ages compared to negative ones, regardless of cultural context [17].

Nevertheless, a threatening environment can influence which emotions of other people are prioritized by children, leading them to judge such emotions as more intense compared with children from a non-threatening environment. Ardizzi et al. show that when reading facial expressions of emotions, the disadvantaged street children are more oriented towards negative emotions [18]. In particular, when comparing two groups, the researchers found that street children more easily identified anger (28.29% versus 7.10% for well-off children) and fear (16% versus 5.3%). In this regard, such children may tend to exaggerate the presence of anger in other people and downplay their positive emotions. McLaughlin states that children in a threatening environment tend to overreact to threats due to their natural reflex of survival in dangerous settings [19]. Similarly, Pollak et al. show that abused children interpret anger as well as well-off children and interpret sadness less accurately (*p* < 0.05) [3].

Still, we do not know of any studies that focus on the war context. It can be assumed, however, that hostilities also present a threat; therefore, they could affect the ability of children with military conflict experience to read anger, sadness, and fear in a similar way.

Hence, our Hypothesis 1 assumes this effect to be present in children from wartime Ukraine:

H1 Children in wartime Ukraine rate anger and fear as more intense than the control group.

### Effects of Threatening Environment on the Accuracy of Emotions Recognition

Threatening environment can also affect the children’s ability to interpret (judge) others’ emotions accurately. By the alteration of judgment we mean, that a facial or body expression signaling one emotion is mistakenly interpreted as a mark of another emotion–a person experiencing (and expressing) joy is, for instance, perceived as angry, etc. Nevertheless, the literature is not unanimous concerning the effect of threats on this type of accuracy.

Scrimin et al. compared emotion recognition in children affected by the Beslan event to a control group, with participants balanced for age and gender. Children with the experience of terrorist attack correctly recognized clearly expressed emotions, such as anger and sadness. The affected children also seemed to label facial expressions showing anger and sadness as anger more often than those from control group, and recognized the clearly expressed emotion of sadness less accurately.

On the other hand, Frankenhuis and de Weerth claim that children who have experienced violence or abuse can recognize even unexpressed, hidden, or distorted aggressive emotions [20]. Pollak et al. have drawn similar conclusions: children who have been abused have a unique and traumatizing social experience, and thus their sensitivity to negative emotions increases [5]. These findings are consistent with those by Scrimin et al. and Frankenhuis and de Weerth [20, 21]. However, according to Bérubé et al., the cognitive processes of abused children is more active, and they react more sharply to negative emotional manifestations [22]. At the same time, the reaction of children with traumatic experiences to emotions is distinctive. Such a child easily recognizes the emotion of happiness but reads anger and fear better, and this skill also remains in adulthood.

Cicchetti takes a different position, noting that the negative emotional experiences that children with traumatic experiences of abuse have been through impair their ability to recognize emotions [23]. In this regard, the existing literature supports the idea that stressors, threatening environments, and experiences of violence and abuse could affect a child’s ability to recognize emotions correctly. At the same time, some conclusions are contradictory. For example, some researchers argue that children who live in a threatening environment or have traumatic experiences are less able to recognize emotions [3, 21]. Other researchers focus on the increased accuracy of children’s recognition of certain emotions [18, 20, 22]. In addition, there are no studies assessing the stressor of war in terms of its effect on children’s emotional development and ability to read emotions. In this regard, it is possible to hypothesize that wartime is also a variant of a threatening environment, which means that it may affect the overall accuracy of children’s recognizing emotion, including their recognition of negative emotions.

Therefore, Hypothesis 2 suggests that this effect is present in children from wartime Ukraine:

H2 Children in wartime Ukraine are, on average, worse at recognizing emotions than the control group but children in wartime Ukraine are better at identifying emotions of anger, sadness, and fear than the control group.

### Present Study

The present study is, to the best of our knowledge, the first attempt to study the peculiarities of emotion recognition by children in war conditions. Our literature review suggests that the effects of wartime can be twofold: (1) affecting the perceived intensity of emotions or (2) affecting the accuracy of recognition. As stated in our hypotheses, we expect the children in wartime Ukraine to judge anger, sadness, and fear as more intense, as observed in earlier studies. However, in general, they will be worse at identifying emotions and their expression compared to the control group of children living in a safe environment in the Czech Republic, whom we have included in the study as a comparison sample because we did not have the opportunity to use a pre-war group in the Ukrainian sample. Based on the results of previous studies, we also hypothesize that aspects of children’s basic emotion recognition are universal under normal conditions, making the Czech control group suitable for comparison. Our study tests these assumptions and provides exploratory findings.

## Methods

The hypotheses and analysis plan were preregistered on OSF (https://osf.io/cpk3b), the data and the analytic script are publicly available in the OSF project (https://osf.io/c6d8x).

### Sample

In total, we analyzed the results from 729 children aged 8–14 years (343 (47%) girls, 386 (53%) boys). See Table 1 for age and country distribution.

**TABLE 1:** Age distribution of the sample (Ukraine, Czech Republic. 2023).

Age	Czech republic	Ukraine	Total
8	12	116	128
9	41	38	79
10	52	105	157
11	54	92	146
12	69	64	133
13	74	3	77
14	8	1	9
Total	310	419	729

The participants in the “wartime Ukraine” group were citizens of Ukraine who lived in the Ukraine territory after the start of the Russian invasion. Most of the participants (83%) attended schools in the Chernihiv region, which was briefly under Russian occupation in March 2022, bordering the invading country. This fact suggests that they were situated in more traumatic war-related settings.

To establish a control group, the experimental tasks were administered to a comparable sample of children living in a country not affected by war nor by similar adverse conditions. The Czech Republic was chosen as a reference country, as it is geographically, culturally, and linguistically close to Ukraine. All participants in the Czech sample attended schools in the South Moravian or Moravian-Silesian regions.

### Procedure and Data Collection

Data were collected using a Qualtrics online survey. We also inquired about the participants’ socio-demographic characteristics and their current emotional state for all basic emotions (happiness, anger, sadness, surprise, fear, and disgust) on a ten-point scale. The total response time did not exceed 20 min.

Children participated voluntarily with the informed consent of their legal guardians. The research plan was approved by the Research Ethics Committee of Masaryk University (EKV-2022-085). In order to ensure safety, the children were surveyed online at home. The participation was anonymous and could be terminated at any time without providing reasons. Due to the ongoing hostilities in Ukraine, it was not feasible to collect a representative sample or ensure that all of the children were impacted in a similar way. We delve deeper into this limit in the Discussion section.

### Measures

As stimuli, we used some items from the Test for identification of socio-emotional deficits [24]. This test is a new diagnostic tool that was developed in years 2018–2022 as a part of a research project funded by the Technological agency of the Czech Republic (project number: TL01000494). The test draws on the Mayer-Salovey-Caruso model of emotional abilities [25], which posits that while emotions are subjective, the capacity to perceive, interpret, and manage them is akin to cognitive abilities. These differences in abilities can be assessed and measured.

The items designed to measure emotion perception consist of photographs and short videoclips. On each item, one actor portrays a specific emotion, mainly via facial expressions, to a lesser degree by body posture and/or body movement. Both photographs and videoclips were taken in a neutral studio setting with monotone light-grey background. The video scenes are not staged, with no setup or prior actions indicating the emotion. Actors include both genders and various ages, though fewer than the total test items, with some appearing in multiple items.

Content-wise, the test builds on the notion of “basic emotions.” This concept was popularized by Ekman, who argued that several emotions and their facial expressions are universally comprehensible across different cultures and may be thus considered as basic, experienced and understood generally by all people, probably on an innate basis [26]. This original list comprised joy, sadness, fear, anger, surprise and disgust. Later, this concept was revised, primarily because the list of basic, universally comprehensible emotions is probably considerably broader (up to 18 different emotions) [27]. However, including a higher number of emotions would markedly increase the time requirements of our design; for this reason, we stuck to the original “basic six” [28].

From the pool of all items comprising the perception part of the Test for identification of socio-emotional deficits, we chose 2 photographs and 2 videoclips for each of the six emotions, establishing 24 items. For each emotion, one photograph and one videoclip portrayed a mild expression, while the second set depicted a more intense expression. Participants viewed 12 photo items first, followed by the remaining 12 videos, in a randomized order.

The participants were tasked with selecting the depicted emotion from a list of basic emotions located below the item. A scroll bar with a 10-point scale was also present in the lower part of the computer screen using which the participants indicated the intensity of the emotion displayed on the item (1 = lowest intensity, 10 = highest intensity).

At the very beginning of the experimental set, before presenting the items, we asked the participants questions regarding their friends, interests, hobbies, etc. Then, they were asked to rate the current intensity of their own emotions (again using a 10-point scale). A separate scale was presented for each emotion of the “basic six” list.

### Data Analysis

The data was analyzed in R (4.1.2) using packages lme4, gorica, restriktor, mirt, performance, and DHARMa [29–33]. Data manipulation was conducted via tidyverse [34]. The confirmatory models were parametrized as a linear mixed model for intensity ratings (given their expected Gaussian distribution) and generalized linear mixed model for accuracy (given its binomial distribution). In both models, we modeled random intercepts for participants (representing their ability) and random intercepts for items (representing their difficulty) as random terms.

After fitting the confirmatory models, we employed informative hypothesis testing which allows to specify order-restricted hypotheses and test their relative likelihood after accounting for their parsimony [35]. In other words, we first specified restriction to the regression coefficients of interest (e.g., for H1 we set the Ukrainian group to have higher average rating of anger and fear intensity, i.e., *b* > 0). Afterwards, the software computes the likelihood of this hypothesis (given the data) and controls for the degree of restriction (with less restricted hypotheses getting more penalty for complexity). Finally, it compares the resulting likelihood to that of an unrestricted hypothesis (i.e., with the maximum likelihood but with the most penalty for complexity because the parameter space was not restricted at all), the complement (i.e., the exact opposite of the specified hypothesis), or another prespecified hypothesis.

## Results

### Descriptives

Means and standard deviations of the item intensity ratings are shown in Table 2 below. Mean difference in intensity ratings between Ukrainian and Czech children was negligible, *d* = 0.04. Item-rest correlations are not reported because the ratings do not assume a reflective measurement model.

**TABLE 2:** Intensity ratings item statistics (Ukraine, Czech Republic. 2023).

	N	M	SD
surprise_1	727	4.30	2.10
anger_1	726	6.40	2.20
fear_1	724	5.30	2.10
disgust_1	726	5.00	2.20
joy_1	726	7.00	2.30
surprise_2	723	6.00	2.30
fear_2	726	6.10	2.30
anger_2	726	7.60	2.20
joy_2	723	7.20	2.40
sadness_1	724	6.40	2.30
disgust_2	721	7.60	2.10
sadness_2	719	5.60	2.30
surprise_3	715	5.10	2.30
fear_3	705	5.50	2.40
joy_3	703	6.50	2.30
fear_4	702	7.10	2.30
anger_3	698	6.30	2.40
surprise_4	701	6.20	2.30
disgust_3	702	7.20	2.20
sadness_3	695	5.70	2.30
anger_4	695	7.10	2.20
joy_4	694	5.70	2.60
sadness_4	689	6.60	2.30
disgust_4	691	6.50	2.10

Interestingly, the children had a strong individual tendency to assess intensity (*ICC* = .32) which was an even stronger predictor than the item characteristics themselves (*ICC* = .12).

Means and item-rest correlations of the emotion recognition items are shown in Table 3. Mean difference in accuracy between Ukrainian and Czech children was small (*d* = 0.15).

**TABLE 3:** Accuracy item statistics (Ukraine, Czech Republic. 2023).

	N	Item-rest r	M
surprise_1	728	.32	0.82
anger_1	726	.20	0.89
fear_1	724	.32	0.86
disgust_1	724	.26	0.84
joy_1	726	.31	0.99
surprise_2	724	.35	0.92
fear_2	726	.00	0,33
anger_2	727	.31	0.94
joy_2	723	.40	0.93
sadness_1	725	.35	0.96
disgust_2	723	.51	0.94
sadness_2	722	.33	0.72
surprise_3	715	.33	0.82
fear_3	706	.18	0.62
joy_3	707	.53	0.98
fear_4	707	.19	0.79
anger_3	700	.18	0.64
surprise_4	706	.24	0.90
disgust_3	703	.57	0.97
sadness_3	698	.39	0.94
anger_4	698	.31	0.94
joy_4	697	.32	0.89
sadness_4	693	.23	0.63
disgust_4	691	.27	0.86

Item characteristics were stronger predictors of accuracy (*ICC* = .27) than participant abilities (*ICC* = .11). We assumed the emotion recognition items would measure a single cluster within Mayer-Salovey-Caruso model, hence the measure is expected to be a reflective, unidimensional construct.

To test this assumption, we fit a *2 PL IRT* model (see De Ayala) which explained the data adequately: M_2_(252) = 562,45, *p* < .001; RMSEA = .043 90%CI [.038 ; .048]; SRMSR = .06; TLI = .87 [36]. On an item level, item fear_2 was problematic due to its low intercorrelation that impacted the IRT estimates (*a* close to zero). Content-wise, the item seemed ambiguous between fear (33%) and disgust (37%). Removing it had no considerable effect on model fit but improved SEs of the item parameter estimates, so we decided to exclude it from further analyses. The accuracy items had borderline reliability (average split-half = .72).

### Intensity Rating Models (H1)

Hereafter, along with the recommendations of, e.g., Mosalves et al., we report the intermediate models we estimated to gradually build the final model [37].

Our baseline model (random item intercepts only) explained a moderate portion of total variance in intensity ratings (total *ICC* = .44). Adding gender and age did not improve the model (χ^2^(2) = 4.71, *p* = .095, ΔAIC = −3, ΔBIC = −21). Including the effect of country, yielded a slight, non-significant, improvement: χ^2^ (1) = 3.78, *p* = 0.052, ΔAIC = 2, ΔBIC = 6. Afixed predictor distinguishing between depictions of fear or anger (1) and others (0), worsened the model: (χ^2^ (1) = 0.53, *p* = .47, ΔAIC = −2, ΔBIC = −1). Finally, we fit the confirmatory model which included all the above and the interaction between country and fear/anger variables, once again worsening the fit: χ^2^ (1) = 0.007, *p* = .93, ΔAIC = −2, ΔBIC = −10.

Therefore, we concluded that our explanatory variables did not explain enough variance in intensity scores. See Table 4 for the final model parameters.

**TABLE 4:** Linear mixed model for intensity ratings (Ukraine, Czech Republic. 2023).

	b	SE	t
intercept	−0.47	0.19	−2.48
gender (1 = boy)	−0.04	0.04	−0.98
age	0.04	0.01	2.62
country (1 = UKR)	0.09	0.05	1.90
type (1 = anger/fear)	0.11	0.16	0,69
country:type	0.002	0.02	0.08

Note: Var(participants) = 0.32, Var(items) = 0.13, Var(e) = 0.56.

**FIGURE 1:**
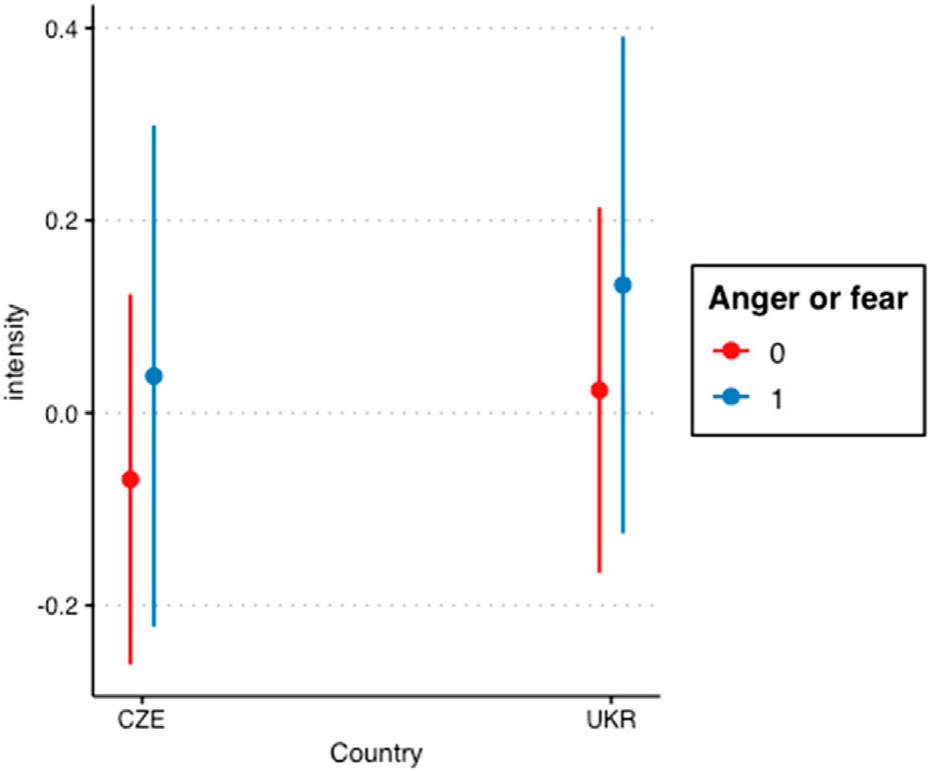
Interaction between country and emotion type - anger and fear versus others (Ukraine, Czech Republic. 2023).

To formally test our hypothesis (H1), we used GORICA to estimate the penalized likelihood of this restriction (i.e., the respective interaction term’s *b* being >0) as opposed to the exact opposite hypothesis (Hc). The H1 was only slightly more supported than Hc (GORICA weights of .501 vs. .499). See Figure 1 for the interaction term visualization.

### Accuracy models (H2)

Our baseline model (same as above) explained a moderate portion of total variance in accuracy ratings (total *ICC* = .38). Including gender and age greatly improved the model fit: χ^2^(2) = 27.40, *p* < .001, ΔAIC = 23, ΔBIC = 7. Adding country as a fixed predictor yielded another noticeable improvement: χ^2^(1) = 33.53, *p* < .001, ΔAIC = 32, ΔBIC = 24. Distinguishing between items depicting fear, sadness or anger (1) and others (0) yielded another improvement in fit: χ^2^(1) = 4.77, *p* = .029, ΔAIC = 2, ΔBIC = 5. Finally, we added the interaction between country and the predictor distinguishing between fear, sadness or anger items and the rest, again noticeably improving model fit: χ^2^(1) = 20.97, *p* < .001, ΔAIC = 19, ΔBIC = 12. In turn, we have found that Ukrainian children recognize positive emotions more accurately than Czech children. See Table 5 for the final model parameters.

**TABLE 5:** Generalized linear mixed model for accuracy (Ukraine, Czech Republic. 2023).

	b	SE	t
(Intercept)	2.82	0.42	6.75
Gender (1 = boy)	−0.25	0.07	−3.51
age	−0.04	0.02	−1.48
Country (1 = UKR)	0.74	0.10	7.54
type (anger/sadness/fear = 1)	−0.81	0.45	−1.80
country:type	−0.45	0.10	−4.67

**FIGURE 2:**
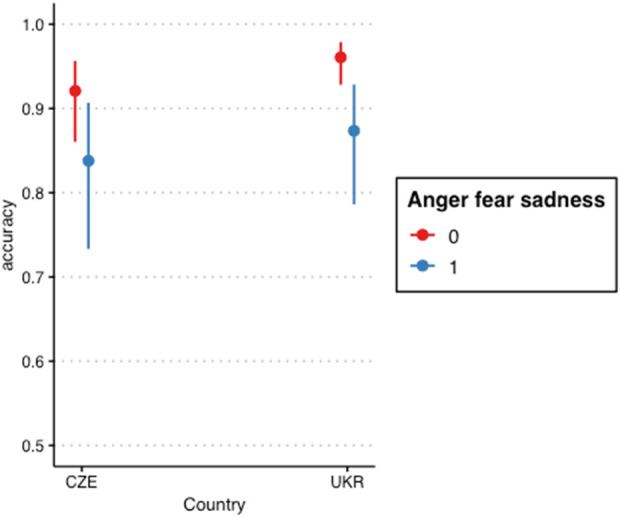
Interaction between country and emotion type - anger or fear or sadness versus others (Ukraine, Czech Republic. 2023).

To formally test H2, we again used GORICA to test the respective interaction term’s *b* being >0 AND the main effect of country being *b* < 0 as opposed to the exact opposite hypothesis (Hc). Unsurprisingly, given both coefficients having the opposite direction in our sample, H2 received no support compared to Hc (0 vs. 1). See Figure 2 for the interaction term visualization.

Fixed terms in neither of the final models exhibited concerning multicollinearity (*VIF* < 2) and there were no concerns regarding the distribution of the residuals or heteroscedasticity.

### Sensitivity Analyses

The effects of children’s current happiness, sadness, fear, and disgust were often significant but not substantial. Only happiness had an effect on intensity ratings (b* = 0.15). Nevertheless, the inclusion of these variables did not change the evaluation of our hypotheses.

## Discussion

This study examined the ability of school-age children to recognize other people’s emotions when exposed to the threats triggered by the war in Ukraine. Previous studies have shown that threatening environment could distort emotional recognition skills. However, wartime should be considered a separate threat factor since individuals facing military conflict are more vulnerable, inclusive of their emotional state, and are more traumatized. Nevertheless, the results surprisingly show that Ukrainian children’s emotional condition and ability to recognize emotions under the influence of threats are discrepant to these expectations. Below, we interpret these results and our exploratory findings.

Our hypotheses implied that the Ukrainian children would exhibit the same pattern as in previous research. However, none of our expectations turned out to be supported by the data. In summary, fear and anger were not rated as more intense than the other emotions (H1). And, perhaps most surprisingly, the Ukrainian children were not less accurate in judging emotions as compared with the control group (H2).

We found that children from both groups were less likely to assess the type of emotions and were more focused on assessing their intensity. However, generally, the ratings of the intensity of emotions in the Ukrainian and Czech groups are almost the same. This fact may indicate that young people in wartime remain attentive and sensitive to the strength of the emotions expressed, which is a natural response [19]. It is also possible to observe some non-typical patterns in the ability of wartime children to recognize the intensity of emotions. In particular, we assumed that it would be more difficult for them to determine the intensity of weak emotions. For the most part, even weak emotions were identified by them as emotions of considerable intensity [18]. The fact that this feature is tracked for all emotions and that recognition of emotion’s intensity does not differ between the Ukrainian and Czech groups, was an interesting and unexpected finding. Accordingly, we may assume that children from Ukraine who go through war experiences do not lose the ability to determine the intensity of fear and anger. Furthermore, children who are exposed to war experiences and are at higher risk of threats seem to identify emotions almost equally well in both static and dynamic manifestations.

Important observations have also been made regarding the accuracy of recognizing emotions by children living in war settings. Initially, it was assumed that children from Ukraine would be worse at recognizing emotions than those in the Czech Republic. However, this hypothesis has not been supported since the results had not revealed significant differences in the accuracy of recognizing all basic emotions between the Ukrainian and Czech groups. For this reason, we can say that hazardous environments and traumatic war experiences do not generally impair a child’s ability to recognize emotions, contradicting the findings by Scrimin et al. and corroborating those by Frankenhuis and de Weerth [20, 21]. At the same time, the recognition of anger, sadness, and fear by Ukrainian children does not differ from that of the Czech group. Earlier studies indicated that traumatic experiences in young people increased their accuracy in recognizing fear and anger [22]. However, comparing the results between the Czech and Ukrainian groups shows that negative emotions appear to be recognized by children living in dangerous and safe conditions with the same accuracy, which radically differs from the findings of previous studies focused on type of risks other than war threats. For example, the current findings are inconsistent with a number of studies, according to which children exposed to threats and abuse and those who find themselves in difficult circumstances are better in interpreting anger and fear and worse in recognizing positive emotions [4, 18–21]. In turn, we have found that Ukrainian children recognize positive emotions more accurately than Czech children. This fact may indicate that war affects emotional recognition in a non-standard way and motivates children to ignore negative impressions and focus on the life’s pleasant moments. We can also assume that war generally does not have a significant effect on emotional recognition in childhood. However, it is important to note that this study did not analyze the severity of traumatic experiences, which could hypothetically influence aspects of emotion recognition. In other words, the difficult environment in which this research project was conducted made it impossible to determine the extent of threats faced by the children and test its relation to emotion recognition accuracy, suggesting that the observations may not apply to the general population of young people living in war settings.

### Limitations of the Study and Future Areas for Research

This study has several limitations. First of all, it did not consider the degree of risk for the participants; that is, it did not assess the children’s proximity to active war zones or the frequency of military threats in their area of residence. In addition, the study did not take into account the characteristics of children’s experiences of war trauma, which could be associated with emotion recognition variables. It also did not examine the quality of life and economic wellbeing of the participants either, although these factors, hypothetically, might also influence the process of emotional recognition. It is also important to recognize that the study results are closely related to age and cultural characteristics and therefore cannot be generalized to the entire population of children who face military threats. Accordingly, we conclude that it is necessary to consider age and culture when assessing the war’s impact on children’s recognizing emotion to gain a deeper understanding of these features. Finally, it is necessary to note that using an online survey might result in some limitations in the accuracy of information collection. This circumstance requires researchers to pay more attention to interpreting the results obtained and involve two or more experts in the assessment process to avoid biases. At the same time, these limitations open areas for future research. In addition to these factors of research interest, it is important to study the effects of the long-term impact of the wartime threats on children’s recognition of emotions. On the face of it, it might seem unlikely that some impairment of emotion perception should manifest itself in the postwar, (and hence peaceful) condition, if it was not found during the obviously more stressful situation of the war itself. However, there are several reasons why it might be so. First, it might provide some additional data about the effects of this type of stress if it lasts for a long time (longer than the span between the start of the war and the realization of the current study). And second, the post-war condition might make it possible to administer a longer set of emotion perception tasks than the one which it was possible to work with now. Such longer and more complex design might permit to find some subtler effects, which could not be detected with a relatively limited number of materials in the present design.
